# Cloning, secretory expression and characterization of recombinant β-mannanase from *Bacillus circulans* NT 6.7

**DOI:** 10.1186/2193-1801-3-430

**Published:** 2014-08-13

**Authors:** Yotthachai Piwpankaew, Supa Sakulsirirat, Sunee Nitisinprasert, Thu-Ha Nguyen, Dietmar Haltrich, Suttipun Keawsompong

**Affiliations:** Interdisciplinary Program in Genetic Engineering, Graduate School, Kasetsart University, Bangkok, 10900 Thailand; Department of Biotechnology, Faculty of Agro-Industry, Kasetsart University, Bangkok, 10900 Thailand; Center for Advanced Studies in Agriculture and Food, Institute for Advanced Studies, Kasetsart University, Bangkok, 10900 Thailand; Department of Food Sciences and Technology, Food Biotechnology Laboratory, BOKU-University of Natural Resources and Life Sciences, Vienna, Austria

**Keywords:** β-mannanase, *Bacillus circulans* NT 6.7, Expression, *Escherichia coli*, Mannooligosacharides

## Abstract

The mannanase gene of *B. circulans* NT 6.7 was cloned and expressed in an *Escherichia coli* expression system. The *B. circulans* NT 6.7 mannanase gene consists of 1,083 nucleotides encoding a 360-amino acid residue long polypeptide, belonging to glycoside hydrolase family 26. The full-length mannanase gene including its native signal sequence was cloned into the vector pET21d and expressed in *E. coli* BL21 (DE3). β-Mannanase activities in the culture supernatant and crude cell extract were 37.10 and 515 U per ml, respectively, with most of the activity in the cell extract attributed to the periplasmic fraction. In contrast, expression of mannanase was much lower when using the *B. circulans* NT 6.7 mannanase gene without its signal sequence. The optimum temperature of recombinant β-mannanase activity was 50°C and the optimum pH was 6.0. The enzyme was very specific for β-mannan substrates with a preference for galactomannan. Hydrolysis products of locust bean gum were various mannooligosaccharides including mannohexaose, mannopentaose, mannotetraose, mannotriose and mannobiose, while mannose could not be detected. In conclusion, this expression system is efficient for the secretory production of recombinant β-mannanase from *B. circulans* NT 6.7, which shows good characteristics for various applications.

## Background

Endo-1,4-β-D-mannanase or β-mannanase (EC 3.2.1.78) is endohydrolase, which catalyzes the random hydrolysis of the β-1,4-D mannopyranosyl linkage in the main chain of mannan-based polysaccharides (Puls 1997). According to the Carbohydrate Active Enzyme database CAZy (http://www.cazy.org), which is based on sequence similarity and hydrophobic cluster analysis, β-mannanase are classified into various families of glycoside hydrolase (GH) i.e., mostly to GH families 5 and 26 and a few members of GH113 (Dhawan and Kaur 2007; Zhang et al. 2008). β-Mannanases are produced by wide range of organisms and it has been widely used in various industrial applications (Araujo and Ward 1990; Dutta et al. 1997; Lee et al. 2003; McCutchen et al. 1996; Puchar et al. 2004). Moreover, there is increasing interest in the application of β-mannanases for the production of mannooligosaccharides (MOS), which have prebiotic properties and thus are of interest for the feed and food industry (Biggs and Parsons 2007; Gibson et al. 2005; Rastall et al. 2005; Smith et al. 2010).

Copra meal is one of the natural sources that contain high amount of galactomannan (Balasubramaniam 1976). In Thailand, 25 million metric ton per year copra meal waste was produced from coconut oil industry (Index Mundi 2014). It has been used as a feed ingredient but its digestibility was limited (Chauhan et al. 2012). Because of the high galactomannan content so the copra meal is an interested substrate for mannooligosaccharides production.

*Bacillus circulans* NT 6.7 was isolated from soil taken from a coconut factory from Nakornpathom province in Thailand. Its β-mannanases could hydrolyze defatted copra meal to mannooligosaccharides which have the prebiotic property making it interesting for industrial applications (Phothichitto et al. 2006). However, the production levels in the wild-type organism are not sufficient for the envisaged applications, and therefore gene expression, by which large amounts of an individual enzyme can be produced in a suitable host strain, is of interest for large scale production and application of this particular enzyme.

*Escherichia coli* is one of most successful expression systems used in biotechnology, showing many advantages for recombinant protein production including well-established and easy operation systems, high yields, and a wealth of molecular tools, to name a few. Recombinant β-mannanases have been successfully produced by *E. coli* systems (Chen et al. 2008; Ethier et al. 1998; Li et al. 2008; Songsiriritthigul et al. 2010; Yang et al. 2009), however, most of these systems were used for intracellular expression and did not success in secretion of the recombinant enzyme.

Our current study shows that recombinant β-mannanases from *B. circulans* NT 6.7, showing high stability and specificity, can be efficiently expressed and secreted in suitable *E. coli-*based expression systems

## Results and discussion

### Cloning and sequencing of the mannanase gene from *B. circulans*NT 6.7

The mannanase gene of *B. circulans* NT 6.7 was obtained by using PCR cloning. Gene sequencing showed that the *B. circulans* NT 6.7 mannanase gene consists of a total 1,083 bp (GenBank Accession No. JF724077)**,** encoding a 360-amino acid polypeptide. The nucleotide sequence of the *B. circulans* NT 6.7 mannanase gene showed the highest identity (99%) with the β-mannanase gene from *Bacillus amyloliquefaciens* strain CICC 23260 (GQ589479.1) and *Bacillus subtilis* strain A33 (DQ269473.1). The amino acid sequence of the *B. circulans* NT 6.7 mannanase showed 99% identity with the β-mannanase from *B. subtilis* (ABB91433.1), and this latter enzyme was classified into glycoside hydrolase family 26 (GH26) based on sequence identity (Altschul et al. 1997). Furthermore, the mannanase sequence was analyzed by SignalP 4.0 (Petersen et al. 2011) and 24 N-terminal amino acid residues (residue number 1–24) were predicted as a signal sequence driving secretion (Petersen et al. 2011).

*B. circulans* NT 6.7 produces an extracellular β-mannanase, which can be employed for several applications (Phothichitto et al. 2006). Even though this enzyme was found to be useful for industrial applications the yields in β-mannanase activity were, however, found to be too low when using the natural source of this enzyme for its production. Hence, heterologous expression was selected for improvement of the production and efficiency of *B. circulans* NT 6.7 β-mannanase. Previous partial sequencing of the mannanase gene from *B. circulans* NT 6.7 β-mannanase showed that it was classified into GH26 family (Lee et al. 2003). This was corroborated by using primers for full-length gene designed from published GH26 mannanase genes of various *Bacillus* spp. This resulted in the successful cloning of the full-length *B. circulans* NT 6.7 mannanase gene, and confirmed that this enzyme in fact belongs to GH26 (Sakulsirirat 2008).

### Expression of the mannanase gene

**Figure 1:**
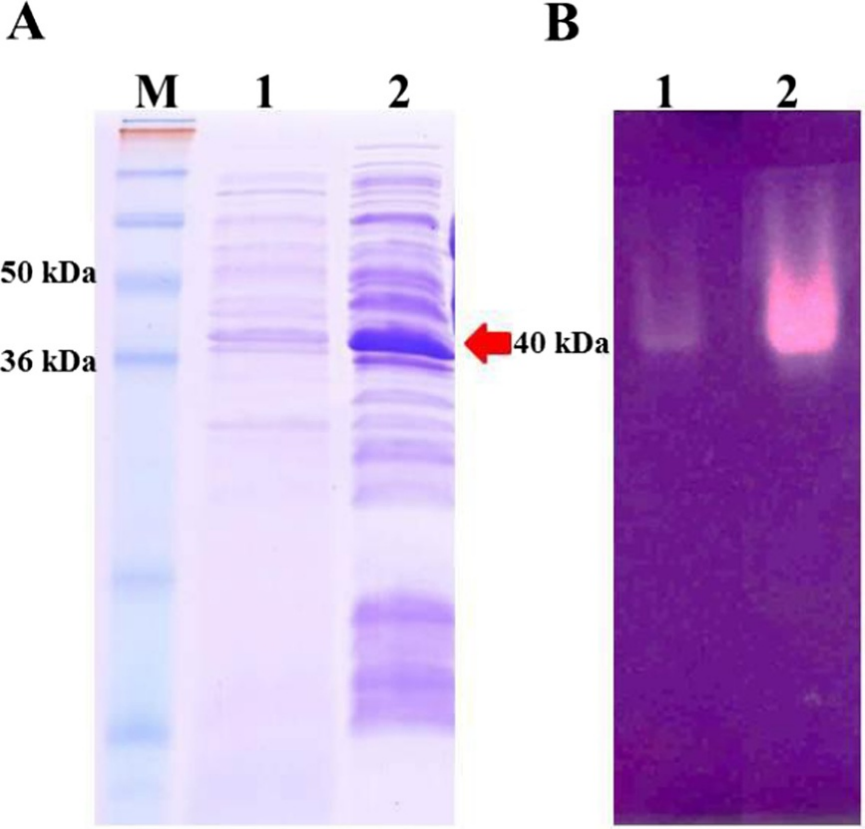
**SDS-PAGE and zymogram analysis of recombinant β-mannanase from** ***E. coli*** **expression system. (A)** SDS-PAGE : lane M, protein marker (Invitrogen, USA); lane 1, crude culture supernatant; lane 2, crude cell lysate. **(B)** zymogram analysis: lane 1, crude culture supernatant; lane 2, crude cell lysate.

The full-length mannanase gene of *B. circulans* NT 6.7 including its native signal sequence was cloned into pET21d, and the gene could be successfully expressed in *E. coli* BL21 (DE3) driven by the T7 RNA polymerase promoter. After induction with various concentrations of IPTG, β-mannanase activity was measured in both culture supernatant and cell lysate fractions. Highest β-mannanase activity was found in cultures that induced with 1.0 mM IPTG, especially in the extracellular fraction, while the level of β-mannanase activity in the cell lysate fractions was similar regardless of the IPTG concentration used. Moreover, intracellular β-mannanase activity decreased after incubation of more than 16 h without the increasing of the extracellular β-mannanase activity. Hence, the optimal expression conditions for mannanase gene expression were induction by 1.0 mM IPTG and 16 h of incubation at 18°C after induction. When using this conditions, *E. coli* BL21 (DE3) harboring pET21d with mannanase gene yielded extracellular and intracellular β-mannanase activities of 37.1 U per ml of crude culture supernatant and 515 U per ml of crude cell extract, respectively, corresponding to a volumetric activity of 37,100 and 515,000 U per liter of culture medium. Most of the intracellular recombinant β-mannanase was found in the periplasmic space of the cells (Table 1). Both intracellular and extracellular β-mannanase showed an identical size that was about 40 kDa on the SDS-PAGE, and also showed activity by zymogram analysis on the substrate gel (Figure 1).

**Table 1:** **The yield of recombinant β-mannanase in different cell compartments of** ***E. coli*** **(from 100 ml culture)**

Cell compartments	Activity	Protein concentration	Specific activity
	(U per ml)	(mg per ml)	(u per mg)
Periplasmic space	497.62	0.03	16,587.33
Cell lysate	45.14	0.05	902.50

The *B. circulans* NT 6.7 mannanase gene without its signal sequence was also cloned into pET21d using the primers Man6.7 F2 (CGGGCCATGGCACACCGTTTATCCCGT) and Man6.7R, and expressed under identical conditions as above. The results obtained showed that recombinant β-mannanase without its signal peptide was hardly secreted regardless of the IPTG concentration used, and intracellular β-mannanase activity was also decreased by a factor of approximately 50, yielding only 9.72 U per ml cell lysate.

The mannanase gene was cloned into the pET21d vector and expressed in *E. coli* BL21 (DE3), a successful standard and commercial expression system (Nguyen et al. 2012). The full-length mannanase gene was cloned into the vector under the T7 promoter without additional signal sequences that are often used in *E. coli* for secretion to the periplasm or extracellular environment (Choi and Lee 2004; Mergulhão et al. 2005). Interestingly, recombinant β-mannanase was secreted into the culture media by this system when induce with appropriate IPTG concentrations, and most of the activity in the fraction termed intracellular was localized in the periplasm, suggesting that the native *Bacillus* signal peptide was recognized by the translocation system of *E. coli* and the recombinant was transported out of the cytosol. We also cloned and expressed the mature mannanase gene without its signal sequence using the same system. The enzyme without signal peptide was not secreted at any IPTG concentration used. In addition, intracellular β-mannanase activity was decreased by more than 50 times, indicating that the efficiency of expression was much lower for this construct. The signal sequence of residues 1 to 24 (**MLKK**LAVCLSIVLLLLGAASP ISA) predicted by SignalP 4.0 (Petersen et al. 2011) shows structural features that have been found as common for other signal sequences as well in that it is composed of a hydrophobic H-domain rich in Ala, Val and Leu, preceeded by a short, positively charged N-domain, which is here shown in bold (Choi and Lee 2004).

This expression system can efficiently drive the production of secreted recombinant β-mannanase from *B. circulans* NT 6.7. Secretory production of an enzyme in *E. coli* offers various advantages including less contamination with host proteins (and hence facilitated purification of the recombinant enzyme), less proteolytic degradation of the target protein and possible increased yield of folded protein because of chaperones found in the periplasm of *E. coli* (Choi and Lee 2004; Mergulhão et al. 2005). Although most of the recombinant β-mannanase was found in the periplasmic space, the enzyme activity of extracellular recombinant β-mannanase alone was almost 20 times higher than that found that of β-mannanase from the natural source *B. circulans* NT 6.7 at the same production scale. Furthermore, overall enzyme activity of this recombinant β-mannanase was about 10 times higher than the yields of recombinant β-mannanase from *B. licheniformis* DSM13, a β-mannanase in the same GH26 family, which was produced with a different *E. coli* expression system using the OmpA signal sequence (Songsiriritthigul et al. 2010). Our results also showed efficient secretion when compared with the recombinant β-mannanase *B. circulans* CGMCC 1416 and *B. circulans* CGMCC 1554, recombinant enzymes from family GH5 that were produced in similar expression systems (Li et al. 2008; Yang et al. 2009). β-Mannanase from *B. circulans* NT 6.7 was classified into GH26 whereas β-mannanase from of *B. circulans* CGMCC 1415 and *B. circulans* CGMCC 1554 were in GH5. The amino acid sequence of β-mannanase was difference between GH26 and GH5. Moreover, there was different in cloned gene including their signal sequences. So, these may cause the different in secretion of the recombinant enzymes from the same expression system.

### Characterization of recombinant β-mannanase

**Figure 2:**
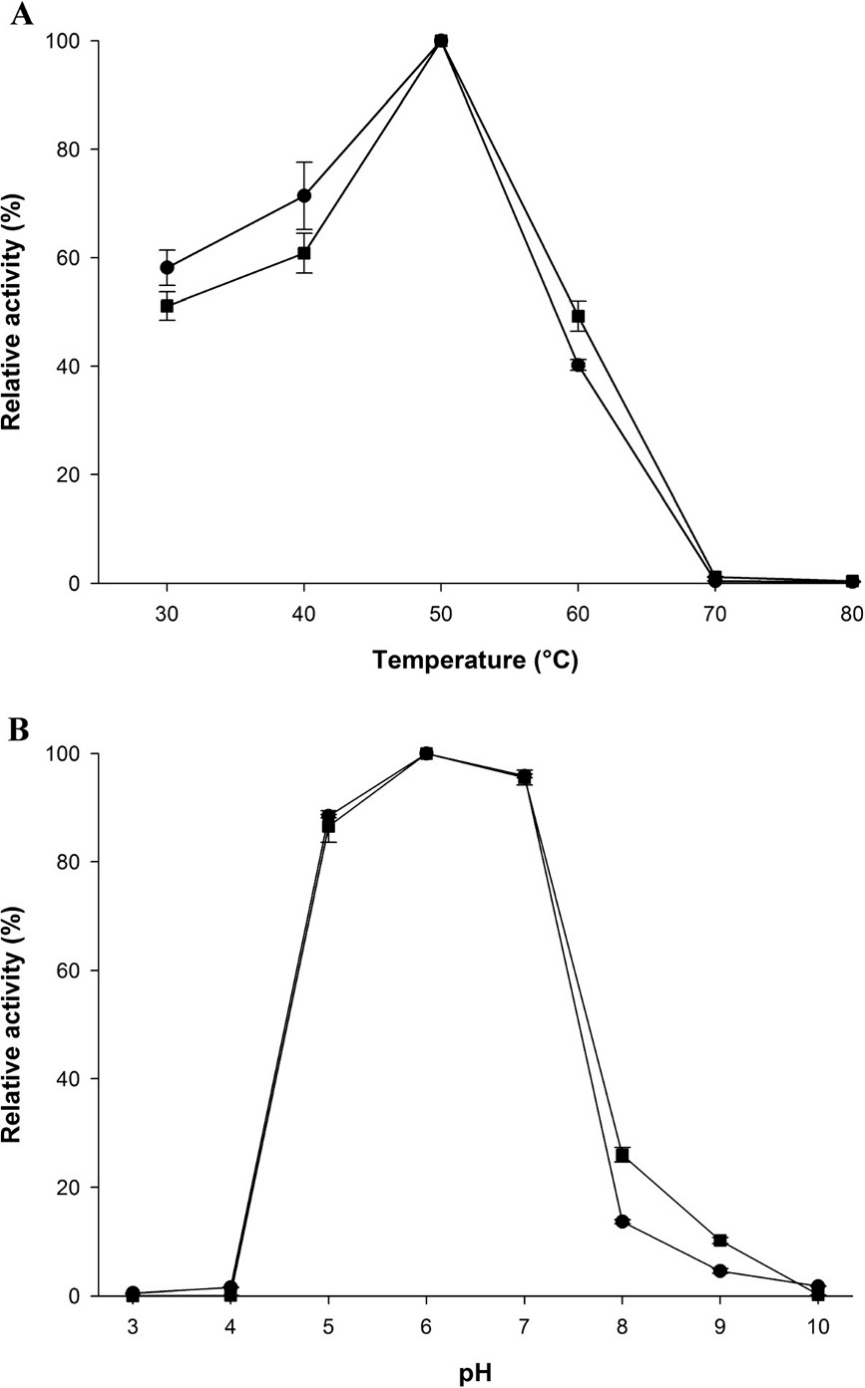
**Effect of temperature (A) and pH (B) on recombinant β-mannanase activity.** Extracellular (–●–) and intracellular (–■–) enzymes were used for these experiment, and results are expressed as relative activity. (100% relative activity of extracellular and intracellular enzymes were 37.10 and 515.00 U per ml, respectively).

The effects of temperature on enzyme activity were determined for both the extracellular and intracellular recombinant β-mannanase. The optimum temperature was measured using the standard assay while varying the temperature from 30°C to 80°C. The optimum temperature of both fractions of the recombinant β-mannanase was 50°C for the 60 minute assay (Figure 2A).

The effects of the pH value on β-mannanase activity were determined for both the extracellular and intracellular recombinant enzyme using the standard assay and varying the pH from 3.0 to 10.0. For both fractions, the optimum pH β-mannanase activity was 6.0, and recombinant β-mannanase was very active in pH range of 5.0-7.0 with more than 80% relative activity (Figure 2B).

The recombinant β-mannanase showed its highest activity on LBG followed by konjac glucomannan. This enzyme also hydrolyzed copra meal and defatted copra meal, other mannan-based substrates from natural product that vary in their degree of substitution with side chains. No activity was found on α-mannan, xylan and cellulose. These results indicate that this recombinant β-mannanase is very specific and exhibits activity only on β-mannan substrates with the preference for galactomannan (Table 2).

**Table 2:** **Substrate specificity of recombinant β-mannanase from** ***B. circulans*** **NT 6.7**

Substrate	Relative activity (%)	
	Extracellular enzyme	Intracellular enzyme
Locust bean gum (LBG)	100.00	100.00
Konjac glucomannan	61.89	68.35
Ivory nut mannan	50.50	53.83
Defatted copra meal	3.26	0.60
Copra meal	0.36	0.15
α-mannan	0.00	0.00
Xylan from oat spelts	0.00	0.00
Carboxymethylcellulose	0.00	0.00

The effect of various metal ions and EDTA on recombinant β-mannanase activity was tested by adding 1 mM of the selected metal ions or EDTA to the enzyme reactions. Their effect on both extracellular and intracellular recombinant β-mannanase activity is shown in Table 3. β-Mannanase activity was slightly enhanced by approximately 5% by Co^+^ ions. Li^+^, Ca^2+^, Mg^2+^, Mn^2+^ and Zn^2+^ ions had minor effects on enzyme activity with the relative activities of more than 90% in the presence of these ions. Cu^2+^ ion had a moderate effect on the enzyme activity, while Fe^2+^ ion and EDTA strongly inhibited β-mannanase activity by 60% and 65%, respectively.

**Table 3:** **Effect of various metal ions and EDTA on the activity of recombinant β-mannanase from** ***B. circulans*** **NT 6.7**

Metal ions/Chemical	Relative activity (%)	
	Extracellular enzyme	Intracellular enzyme
Control	100.00	100.00
Co^+^	104.74	106.32
Ca^+^	95.45	97.29
Zn^2+^	97.50	92.24
Mn^2+^	95.26	95.65
Mg^2+^	94.32	95.46
Li^+^	96.82	97.70
Cu^2+^	79.05	64.08
Fe^2+^	40.12	39.18
EDTA	32.00	37.95

For the hydrolysis of LBG, both of extracellular and intracellular recombinant β-mannanase showed an identical hydrolysis pattern. After 6 h of hydrolysis, 10 mg LBG was hydrolyzed to be various mannooligosaccharide products consisting of mannohexaose, mannopentaose, mannotetraose, mannotriose and mannobiose, with mannotriose as the main reaction product (Table 4). No mannose was formed under these conditions, and even when extending the incubation time to 24 h mannose could not be detected in the hydrolysis mixture.

**Table 4:** **Mannooligosaccharide products of LBG hydrolysis with recombinant β-mannanase from** ***B. circulans*** **NT 6.7 at 6 h of incubation**

Mannooligosaccharides	Amount of hydrolysis product (mg)	
	Extracellular enzyme	Intracellular enzyme
Mannohexaose (M6) and mannopentaose (M5)	0.063	0.051
Mannotetraose (M4)	0.109	0.149
Mannotriose (M3)	0.346	0.376
Mannobiose (M2)	0.237	0.288

HPLC analysis of hydrolysis products of defined mannooligosaccharide substrates showed that the recombinant β-mannanase hydrolyzed mannohexaose, mannopentaose and mannotetraose into smaller molecules but was not active on mannotriose and mannobiose (Table 5). Mannohexaose and mannopentaose were hydrolyzed to be mannotetraose, mannotriose, mannobiose after 0.5 h of incubation and even mannose formed after an extended incubation for 6 h. At 6 h of incubation, almost 90% of mannohexaose and 70% of mannopentaose were hydrolyzed. Moreover, the recombinant enzyme hydrolyzed mannotetraose into smaller oligomers including mannose after 6 h of incubation, and the concentration of these products further increased after 24 h of incubation.

**Table 5:** **Mannooligosaccharide products of the hydrolysis of mannohexaose, mannopentaose and mannotetraose with recombinant β-mannanase from** ***B. circulans*** **NT 6.7 at 6 h of incubation**

Substrates	Amount of hydrolysis product (mg)					
	M1	M2	M3	M4	M5	M6
Mannohexaose (M6)	0.048	0.995	1.207	1.517	0.000	-
Mannopentaose (M5)	0.068	1.148	1.475	0.532	-	-
Mannotetraose (M4)	0.040	0.074	0.089	-	-	-

Both of extracellular and intracellular recombinant β-mannanase from *B. circulans* NT 6.7 showed the same characteristics including protein size, temperature and pH optima for activity, substrate specificity, effects of ion on activity as well as the hydrolysis patterns obtained after LBG hydrolysis. Moreover, the recombinant, heterologously produced β-mannanase also has the same enzyme properties as the wild type enzyme (Pangsri 2014). The temperature and pH optima of both of wild type and recombinant β-mannanase from *B. circulans* NT 6.7 were 50°C and 6.0. Both of recombinant and wild type enzyme was active on various mannan substrates especially LBG, a high-molecular-mass galactomannan. The results of substrate specificity showed that this recombinant enzyme is specific for the hydrolysis of β-1,4-mannosyl linkages without activity on structurally related polysaccharides such as xylan or cellulose as same as wild type β-mannanase from *B. circulans* NT 6.7. There were the difference in enzyme properties such as the optimum temperature and pH, enzyme stability and also specificity in both of native and recombinant β-mannanases that have characterized from various organisms depended on the source of enzymes. This recombinant β-mannanase from *B. circulans* NT 6.7 had high enzyme activity at the appropriate conditions for industrial application. It also had high specificity on mannan with the preference for galactomannan that was very advantage on the mannooligosaccharide production.

HPLC analysis of hydrolysis products confirmed that recombinant β-mannanase from *B. circulans* NT 6.7 hydrolyzes the mannan backbone in a random fashion, releasing oligosaccharides of different size, and mannooligosaccharides of two to six mannose units could be identified after LBG hydrolysis, while no free mannose was detected by HPLC analysis. Various mannooligosaccharide substrates (M4 to M6) were again cleaved in a random fashion, since a range of different products was observed. The smaller mannooligosaccharides M3 and M2 when used as pure substrates were not hydrolyzed by the enzyme, indicating that the active site must accommodate at least four mannose units for catalysis. Altogether, these results suggested that this this recombinant β-mannanase had the effective hydrolysis on various β-mannan substrates as wild type enzyme from *B. circulans* NT 6.7 which could be used for the production of prebiotic mannooligosaccharides from natural resources of high galactomannan content such as copra meal or LBG.

## Conclusions

This study has shown an efficient expression system for the secretory production of β-mannanase gene from *B. circuans* NT 6.7. Furthermore, the recombinant β-mannanase has properties including its optimum temperature and pH, high stability and the effective hydrolysis of mannan substrates that are promising for large-scale production.

## Methods

### Bacterial strains, plasmids and media

*B. circulans* NT 6.7 was cultured in Luria-Bertani (LB) medium at 37°C with shaking at 200 rpm for isolation of genomic DNA. *E. coli* DH5α and *E. coli* BL21 (DE3) were used as molecular cloning host and expression host, respectively. *E. coli* strains were cultured in LB medium containing 100 μg/ml ampicillin at 37°C with shaking at 125 rpm. The plasmid pGEM-T Easy (Promega, USA) was used for cloning and sequencing experiments, and the plasmid pET-21d(+) (Novagen, Germany) was used as expression vector in *E. coli* BL21 (DE3).

### Construction of recombinant plasmids

Genomic DNA of *B. circulans* NT 6.7 was extracted by using the illustra™ bacteria genomicPrep Mini Spin Kit (GE Healthcare, UK). Primers F1 (ATGCTTAAAAAGTTAGC AGTCTGYCT) and R1 (TTATTCCGCGATCGGCGTCAA) were designed based on the conserved nucleotide sequence of β-mannanase from *Bacillus* GH26. The amplification was performed by using Dream® *Taq* DNA polymerase (Fermentas, USA) and the reaction cycle consisted of initial denaturation at 95°C for 5 min followed by 30 cycles of 95°C for 1 min, 55°C for 1 min and 72°C for 2 min, and the final extension at 72°C for 5 min. The amplification product was ligated into the pGEM-T Easy vector and transformed into *E. coli* DH5α for sequencing.

Primers Man6.7 F (GCGGCCATGGCTATGCTTAAAAAGTTAGCA) with the *Nco*I recognition site and Man6.7R (CCGGCTCGAGTTCCGCGATCGGCGT) with the *Xho*I recognition site were designed based on the result of sequencing analysis. The PCR reaction contained approximately 100 ng of *B. circulans* NT 6.7 genomic DNA as template, 10 pmol of each primer, 0.2 mM each dNTP, 2.5 mM MgCl_2_, 1× buffer and 1U Phusion High-Fidelity DNA polymerase (New England Biolabs, UK). Amplification condition consisted of initial denaturation at 98°C for 3 min followed by 30 cycles of 98°C for 30 s, 60°C for 30 s and 72°C for 45 s, and the final extension at 72°C for 5 min. The amplification product was purified by using the illustra™ GFX PCR DNA and Gel Band Purification Kit (GE Healthcare, UK) and digested with *Nco*I and *Xho*I restriction enzymes. The digestion product was ligated into the *Nco*I-*Xho*I site of pET21d and subsequently transformed into *E. coli* BL21 (DE3). Positive clones were confirmed by colony-PCR and sequencing.

### Expression of mannanase

For the recombinant expression of the mannanase gene, overnight cultures of *E. coli* BL21 (DE3) carrying the respective expression plasmid were inoculated into 100 ml of LB medium with 100 μg/ml of ampicillin. The cultures were incubated at 37°C and 200 rpm until the OD600 reached 1.0 and then induced with adding IPTG to a final concentration of 0.1, 0.5 and 1.0 mM. After IPTG induction, the cultivation was carried out at 18°C with 200 rpm for 16, 18 and 20 h. Both of culture supernatant and cell pellet were collected after cultivation. Cells were harvested by centrifugation at 8,000 rpm, 4°C for 30 min. The cell pellet was washed twice with 50 mM potassium phosphate buffer pH 6.0 and then resuspended in the same buffer. Cells were disrupted three times by glass bead stirring with 5000 rpm for 30 seconds. Cell-free extracts were obtained by centrifugation at 13,000 rpm for 30 min and used for subsequent analysis.

### Enzyme assay and protein determination

The standard β-mannanase activity assay consisted of 100 μl of 1% locust bean gum (LBG) in 50 mM potassium phosphate buffer pH 6.0 and 100 μl of enzyme solution. The reaction mixture was incubated at 50°C for 60 min. The amount of reducing sugar released was subsequently determined by the dinitrosalicyclic acid (DNS) method using D-mannose as the standard (Miller 1959). One unit (U) of β-mannanase activity was defined as the amount of enzyme producing 1 μmol of mannose equivalents per minute under the assay conditions.

Protein concentrations were determined by the Bradford method using Protein Assay Reagent (BioRad, Austria) with bovine serum albumin as the standard.

### Protein localization, protein electrophoresis and zymogram analysis

For localization of recombinant β-mannanase within the cell, the periplasmic and the cell lysate fraction were separated following the method by Songsiriritthigul et al*.* (2010). For extraction of the periplasmic content, cells were resuspended in spheroplast buffer [100 mM Tris–HCl, pH 8.0, 0.5 mM EDTA, 0.58 M sucrose, and 20 μg/ml phenylmethylsulfonyl fluorides (PMSF)]. After incubation for 5 min on ice, cells were collected and re-suspended in water containing 1 mM MgCl_2_. The supernatant was then collected by centrifugation as the periplasmic fraction. Subsequently, cells were washed and resuspended in lysis buffer (50 mM Tris–HCl and 0.5 mM EDTA). After centrifugation, the supernatant was collected as the cell lysate fraction. Proteins in each fraction were analyzed by protein electrophoresis.

Sodium dodecyl sulfate polyacrylamide gel electrophoresis (SDS-PAGE) was performed using 15% (w/v) polyacrylamide gels. Protein bands were visualized by coomassie brilliant blue staining.

Zymogram analysis of recombinant β-mannanase was performed by a gel activity assay using LBG as substrate. Both of extracellular and intracellular recombinant fractions were run on 15% (w/v) native polyacrylamide gels. After electrophoresis, the native gel was placed on a substrate gel and incubated at 50°C for 1 h. Then, the substrate gel was stained with Congo red solution, destained with 1 M sodium chloride and background stained with 5% acetic acid. Enzyme activity on the substrate gel was visualized as a clear zone against a blue background.

### Effect of temperature and pH on β-mannanase activity

The optimum temperature of β-mannanase activity was determined using the standard assay with 1% LBG in 50 mM potassium phosphate buffer pH 6.0 as substrate at the temperature range of 30-60°C.

The optimum pH of β-mannanase activity was determined using the standard assay with 1% LBG in the pH range of 3.0-10.0 at the 50°C.

### Substrate specificity

The substrate specificity of recombinant β-mannanase was examined by measuring the enzyme activity under standard assay condition using various substrates including LBG, konjac glucomannan, ivory nut mannan, copra meal, defatted copra meal, α-mannan from yeast cell walls, xylan from oat spelts and carboxymethylcellulose (CMC).

### Effect of various metal ions and EDTA on β-mannanase activity

The effect of metal ions on β-mannanase activity was determined by measuring the activity of the enzyme in the presence of various metal ions including Li^+^, Ca^2+^, Cu^2+^, Fe^2+^, Mg^2+^, Mn^2+^, Zn^2+^ and Co^+^, each at final concentration of 1 mM using standard assay condition. EDTA was employed at a similar concentration.

### Analysis of hydrolysis products of recombinant β-mannanase

For the hydrolysis study, 1% of LBG and the 1% of mannooligosaccharides mannobiose, mannotriose, mannotriose, mannotetraose, mannopentaose and mannohexaose were hydrolyzed by recombinant β-mannanase. In the hydrolysis reaction, 1 ml (10 mg) of substrates were incubated with 10 U of recombinant enzyme in 50 mM potassium phosphate buffer pH 6.0 (2 ml of total volume) at 50°C for 0, 0.5, 1, 3, 6, 12 and 24 h. The reactions were stopped by boiling for 5 min. The hydrolysis products were then analyzed by high pressure liquid chromatography (HPLC) using an Aminex-HPX42C column (Bio-rad, USA) with authentic mannooligosaccharides (Megazyme, Ireland) as standard.
